# Detection and Genetic Correlation Analysis of Diarrhea Cases and Norovirus in Oysters in Yantai, China

**DOI:** 10.3389/fpubh.2022.819890

**Published:** 2022-05-25

**Authors:** Zhenlu Sun, Peihua Niu, Miao Jin, Qiao Gao, Ji Wang, Xuejun Ma

**Affiliations:** ^1^NHC Key Laboratory of Medical Virology and Viral Diseases, National Institute for Viral Disease Control and Prevention, Chinese Center for Disease Control and Prevention, Beijing, China; ^2^Yantai Center for Disease Control and Prevention, Yantai, China; ^3^Department of Viral Diarrhea, National Institute for Viral Disease Control and Prevention, Chinese Center for Disease Control and Prevention, Beijing, China

**Keywords:** norovirus, oyster, diarrhea, coastal areas, genotype, homology, phylogenetic analysis

## Abstract

**Background:**

This study aimed to assess the correlation between Norovirus (NoV), diarrhea, and raw oysters from the eastern coastal areas of Yantai, Shandong, China.

**Methods:**

Marine oysters were selected from the three aquatic markets in Laishan district, Yantai City, in March 2019. Meanwhile, 100 fecal samples were collected from patients with diarrhea from the same areas during the same period. Nucleic acids were extracted from these samples and detected by employing reverse transcription polymerase chain reaction (RT-PCR) for NoV GI/GII. The VP1 gene of the coat protein of NoV was amplified by semi-nested RT-PCR and sequenced. Sequence comparison of VP1 was performed with BioEdit software, and the evolutionary tree was constructed with Mega7.0 software.

**Results:**

Of the 151 oysters, 42 (27.8%) were positive for NoV. Among them, 32 (21.2%) were GII-positive, 10 (6.6%) were GI-positive, and one GI VP1 sequence was obtained in the oyster samples. Of 100 fecal samples from patients with diarrhea, 38 were GII-positive and 17 were GI-positive. Totally, 19 GII VP 1 sequences and eight GI VP 1 sequences were obtained. Two G1 VP 1 sequences in two fecal samples showed 98.7% nucleotide sequence identity and 99.1% amino acid sequence identity G1 VP 1 acquired in the oyster sample.

**Conclusions:**

The results suggest that oysters may be responsible for the spread of NoV in Yantai, Shandong province, China.

## Introduction

Norovirus (NoV) is an important pathogen causing sporadic non-bacterial gastroenteritis. About 90% of acute gastroenteritis and 50% of foodborne gastroenteritis outbreaks worldwide are caused by NoV (1). NoV is an RNA virus with a 7.5–7.7 kb genome that consists of three open reading frames (ORF). ORF1 encodes non-structural proteins, including RNA-dependent RNA polymerase (RdRp), nucleoside triphosphatases (NTPases), and proteases; ORF2 and ORF3 encode the major capsid protein VP1 and the minor capsid protein VP2, respectively. In 2019, Chhabra et al. (2) further classified and updated the toxic strains of NoV and expanded the number of genogroups to 10 (GI-GX) and the number of genotypes to 49.

Norovirus is generally transmitted *via* food, sick people, and contaminated surfaces. Person-to-person transmission is the dominant transmission route, while other routes may include transmission through food and water. Human or animal excrement is discharged offshore through rainwater, sewage, etc. Human NoV is the most common cause of epidemic gastroenteritis following the consumption of bivalve filter-feeding shellfish (3). Offshore cultured bivalve filter-feeding shellfish, including oysters, can filter large amounts of plankton in seawater through their gills to obtain nutrition and, at the same time, enrich harmful pathogens in seawater.

Previous studies (4, 5) have shown that the concentration of NoV and rotavirus in the shellfish digestive system is significantly higher than that found in seawater (6). Japanese scholars were the first to explain that NoVs can migrate in the population through oysters in a similar geographical area (7). Later, several studies in Europe and Japan also mentioned the correlation between NoV pollution in shellfish and NoV infection in the population (8–10). However, there are few relevant reports in China. More evidence is still needed to investigate the transmission path of NoVs in the population and environment.

Yantai is a coastal city located in eastern China, northeast of the Shandong Peninsula, with a coastline of 909 km and 63 islands. Yantai faces the Liaodong Peninsula, South Korea, and Japan across the sea. The outbreak of NoV in the coastal city is higher than that in the inland areas. In this study, we collected 151 seafood oysters from the three aquatic product markets in Lai Shan, eastern coastal areas of Yantai, and 100 feces from patients with diarrhea in the same areas during the same period. We then analyzed the correlation between NoV in oysters and human feces in this area.

## Materials and Methods

### Sample Collection

From 12 to 13 March 2019, seafood oysters were selected from the three aquatic products markets in Lai Shan district, eastern coastal areas of Yantai City. To enrich oyster NoV according to the size of oysters, the sampling specifications of each sample were divided into 3–4 oysters/part (larger oysters) and 5–7 oysters/part (smaller oysters), about 200 g/part, counting 151 samples. Samples sent for inspection the same day after collection were refrigerated and transported at 0–5°C; samples that could not be tested the same day after arriving at the laboratory were frozen in a low-temperature refrigerator at −80°C. From 14 to 16 March 2019, 100 fecal samples were collected from patients with diarrhea (loose, watery, sticky liquid or pus, and blood stool for more than three times in 24 h) in nearby areas during the same period. The study was approved by the Second Ethics Review Committee of the National Institute for Viral Disease Control and Prevention, Chinese Center for Disease Control and Prevention. All patients read and signed informed consent.

### Oyster NoV Detection Method

#### Oyster Sample Pretreatment

Oyster shell dirt was washed off with running tap water. Then, a double oyster shell was opened according to a previously described approach (11, 12), and the digestive gland tissue was dissected with sterilized scissors and tweezers and placed in a sterile plate.

#### Enrichment of Oyster NoV

Five grams of oyster digestive glands were cut into pieces (11, 12), put into a 50-ml centrifuge tube, and ground with a grinding pestle until homogeneous. Then, 2 g of samples were weighed and placed in a 50-ml centrifuge tube, mixed with 2 ml of phosphate buffer (PBS) and 10 μl of proteinase K solution, shaken for 60 min at 37°C, 280 revolutions/min, and placed in a water bath at 60°C for 15 min. Finally, the samples were centrifuged for 5 min at 3,000 revolutions/min, and the supernatant was taken in a new 1.5-ml EP tube.

#### Extraction and Detection of Oyster Virus Nucleic Acid

The precipitate was fully dissolved in 280-μl of PBS, and the virus RNA was extracted by the QIA amp Viral RNA Mini kit, following the manufacturer's instructions; the final nucleic acid elution volume was 60 μl. NoV GI/GII group real-time fluorescence reverse transcription-polymerase chain reaction (RT-PCR; Jiangsu Bioperfectus Technologies, China) nucleic acid detection kit was used for virus detection, and negative, positive, and blank controls were established. The cyclic threshold value (Ct value) ≤ 38 and a typical “S” curve were judged as positive, indicating that NoV nucleic acids were detected in the samples. Positive nucleic acids were stored at −80°C for subsequent tests.

#### Detection of Oyster NoV Using Semi-nested RT-PCR

For NoV-positive nucleic acid, VP1 region genes of the NoV GI and GII capsid protein were amplified by a semi-nested RT-PCR reaction according to previous studies (13, 14). A one-step RT-PCR kit (Shanghai BioGerm Medical Technology Co.) was used for one round of amplification and Amplitaq Gold 360 Master Mix for two rounds of amplification. The primer sequences are shown in Table 1. The first amplification system (50 μl) included: 5 × 10.0 μl buffer, 10 mmol/L of 2.0 μl dNTPs, and 1.0 μl of upstream and downstream primers (33 μmol/L). Enzyme Mix 2.0 μl, RNase-free water 29.0 μl, template 5.0 μl; the first reaction condition was: reverse transcription at 50°C for 30 min; pre-denaturation at 95°C for 15 min; denaturation at 94°C for 30 s, annealing at 50°C for 30 s, extension at 72°C for 1 min, 30 cycles; stretching at 72°C for 7 min, and storage at 4°C. The second amplification system (50 μl) was as follows; 2 × Mix 25.0 μl, 1.0 μl of upstream and downstream primers (33 μmol/L), RNase-free water 21.0 μl, one round of product 2.0 μl; the second reaction condition was: pre-denaturation at 95°C for 10 min; denaturation at 95°C for 30 s, annealing at 50°C for 30 s, extension at 72°C for 1 min, 35 cycles; stretching at 72°C for 7 min and storage at 4°C. The target fragments of GI and GII groups, which were 330 and 387 bp, respectively, were using automatic capillary electrophoresis. Positive electrophoresis samples were sent to Sangon Biotech for nucleic acid sequencing.

**Table 1:** Primer sequences of Norovirus (NoV) in a semi-nested reverse transcription polymerase chain reaction (RT-PCR).

**Primer**	**First-round sequence (5^′^-3^′^)**	**Second-round sequence (5^′^-3^′^)**
Norovirus GI	COG1F: CGYTGGATGCGITTYCATGA	G1SKF: CTGCCCGAATTYGTAAATGA
	G1SKB: CCAACCCARCCATTRTAGA	G1SKR: CCAACCCARCCATTRTACA
Norovirus GII	COG2F: CARGARBCNATGTTYAGRTGGATGAG	G2SKF: CNTGGGAGGGGGATCGCAA
	G2SKB: CCRCCNCCATRHCCTRRTRACAT	G2SKB: CGRCCNGGATRHCCRTTRTACAT

#### Extraction of Viral RNA and Detection of NoV in Human Diarrhea Fecal Samples

About 100 frozen fecal samples from patients were dissolved in 0.9 ml of saline and placed in a 1.5-ml EP tube. Then, 0.1 ml of liquid fecal specimen was added to prepare a 10% fecal suspension, vortexed three times, and centrifuged for 5 min at room temperature at 3,000 revolutions/min. The supernatant was then absorbed for nucleic acid extraction (15). For nucleic acid extraction, the QIAamp Viral RNA Mini kit nucleic acid extraction kit (Qiagen, Germany) was used according to the instructions for RNA extraction. All samples were detected by the NoV (GI and GII groups) real-time fluorescence RT-PCR detection kit (Jiangsu Bioperfectus Technologies, China), the CT ≤ 38, and the curve is s-shaped, which is judged as positive, the CT >40 is judged as negative. All experiments were carried out using 7500 Fast Real-Time PCR systems (AppGliedBiosystems, USA). NoV types I and II were qualitatively detected. The detection method was performed according to the kit instructions.

#### Amplification and Sequencing

Norovirus capsid protein region-specific oligonucleotide primers, COG2F: 5′-CARGARBCNATGTTYAGRTGGATGAG-3; G2-SKR:5′-CCRCCNGCATRHCCRTTRTACAT-3′ were used. TaKaRa one-step RNA PCR kit was used. The reaction conditions were: incubation at 50°C for 30 min; denaturation at 94°C for 2 min; denaturation at 94°C for 30 s, annealing at 42°C for 30 s, and extension at 60°C for 45 s, a total of 30 cycles; the last 72°, extended by 10 min. The amplified fragments were confirmed by the electrophoresis products according to the DNA standard molecular weight (Marker) position. Primer sequences were synthesized by Sangon Biotech, and positive electrophoresis samples were sent to Sangon Biotech for nucleic acid sequencing.

### Genetic Sequencing Analysis

Sequencing results were analyzed with BioEdit software and spliced with SeqMan. Blast was used to retrieve the reference sequence of the spliced sequence in GenBank. A phylogenetic tree based on the VP1 gene was drawn by the neighborhood connection method in Mega 7.0. The bootstrap value was set at 1,000 replicates, and phylogenetic analysis was conducted.

Sequencing results were analyzed with BioEdit software and spliced with SeqMan. Blast was used to retrieve the reference sequence of the spliced sequence in GenBank. A phylogenetic tree based on the VP1 gene was drawn by neighbor-joining with Mega 7.0 and the Kimura two-parameter model was selected. The tree topology was re-evaluated using Bootstrap method with 1,000 replicates.

## Results

### Detection of NoV in Oysters in the Yantai Area

In March 2019, 151 oysters were collected from the Yantai local sea area, among which 42 were positive to NoV nucleic acid by RT-PCR, achieving a positive rate of 27.8%. Among these, 32 samples were GII-positive, and the positivity rate was 21.2%. As shown in Table 2. Moreover, 10 GI samples were positive, achieving a positivity rate of 6.6%. Because of low viral loads in most positive samples, only one GI VP1 sequence was successfully obtained by sequencing.

**Table 2:** Detection of NoV in oyster samples.

**Type**	**Number of positive samples**	**Positive percentage**
Norovirus GI	10	6.6%
Norovirus GII	32	21.2%

### Detection of NoV in Human Diarrhea Fecal Samples in the Yantai Area

About 100 fecal samples were collected from patients with diarrhea between March and April 2019. About 38 samples were GII-positive, and 17 samples were GI-positive by RT-PCR. Totally, 19 GII VP1 sequences and eight GI VP1 sequences were successfully obtained. As shown in Table 3. Eight GI-positive samples were numbered as 05, 06, 13, 14, 43, 44, 88, and 89, respectively.

**Table 3:** Detection of NoV in human diarrhea samples.

**Type**	**Number of positive samples**	**Positive percentage**
Norovirus GI	17	17%
Norovirus GII	38	38%

### Genotyping and Phylogenetic Analysis of the VP1 Region of Oyster NoV

**Figure 1:**
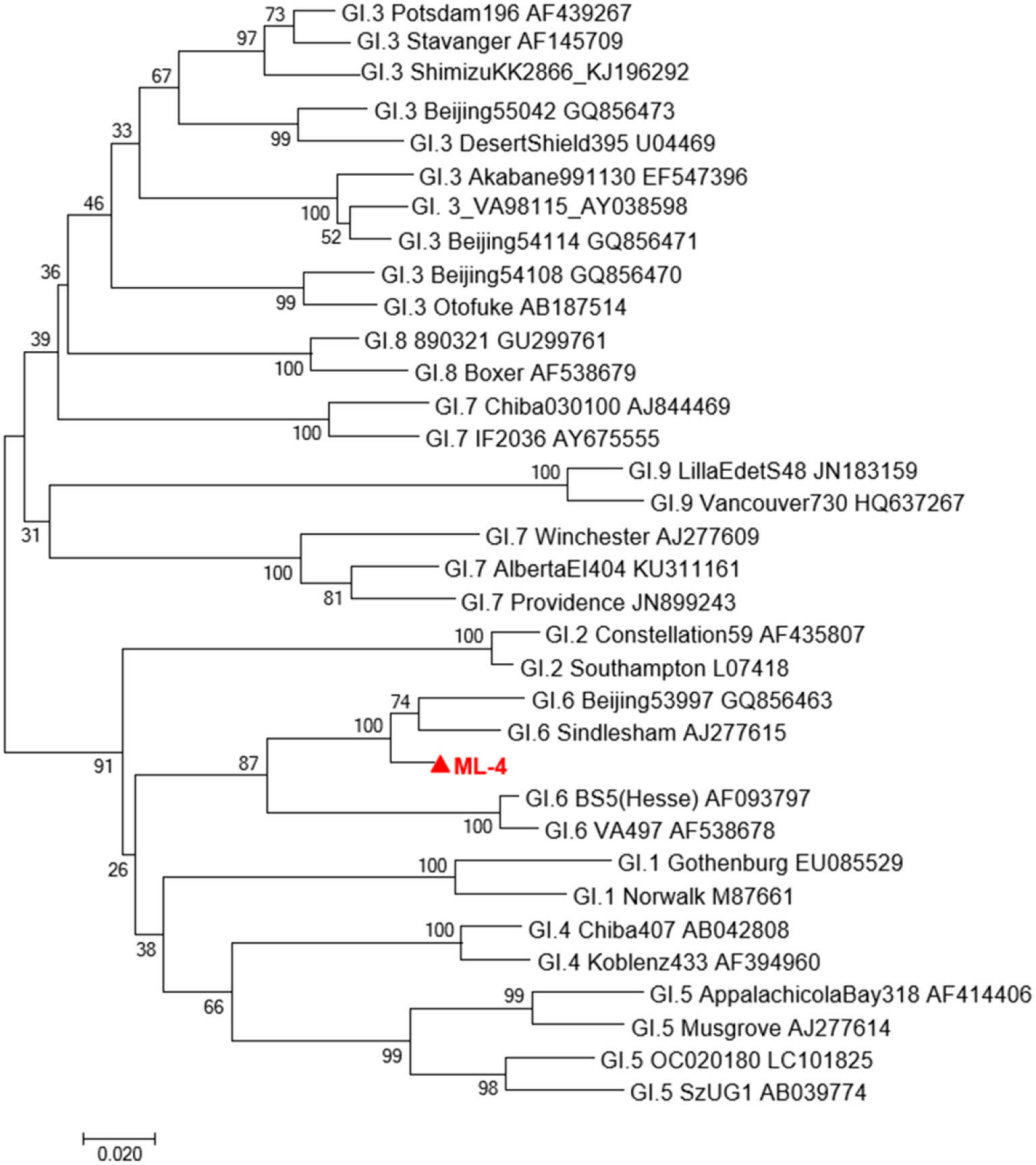
A phylogenetic tree of GI type sequence in Oyster isolates in Yantai city and representative GI strains of other genotypes. Virus strains indicated by 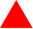 are from Yantai.

Sequencing results of ML04 samples were compared with GI sequences published in GenBank by Blast, and a phylogenetic tree was constructed. As shown in Figure 1, the evolutionary tree graph further confirmed that ML04 belongs to the GI.6 type and has the highest homology with GI.6 Sindlesham AJ277615.

### Phylogenetic Analysis of Oyster NoV GI VP1 Sequence and Human NoV GI VP 1 Sequence

**Figure 2:**
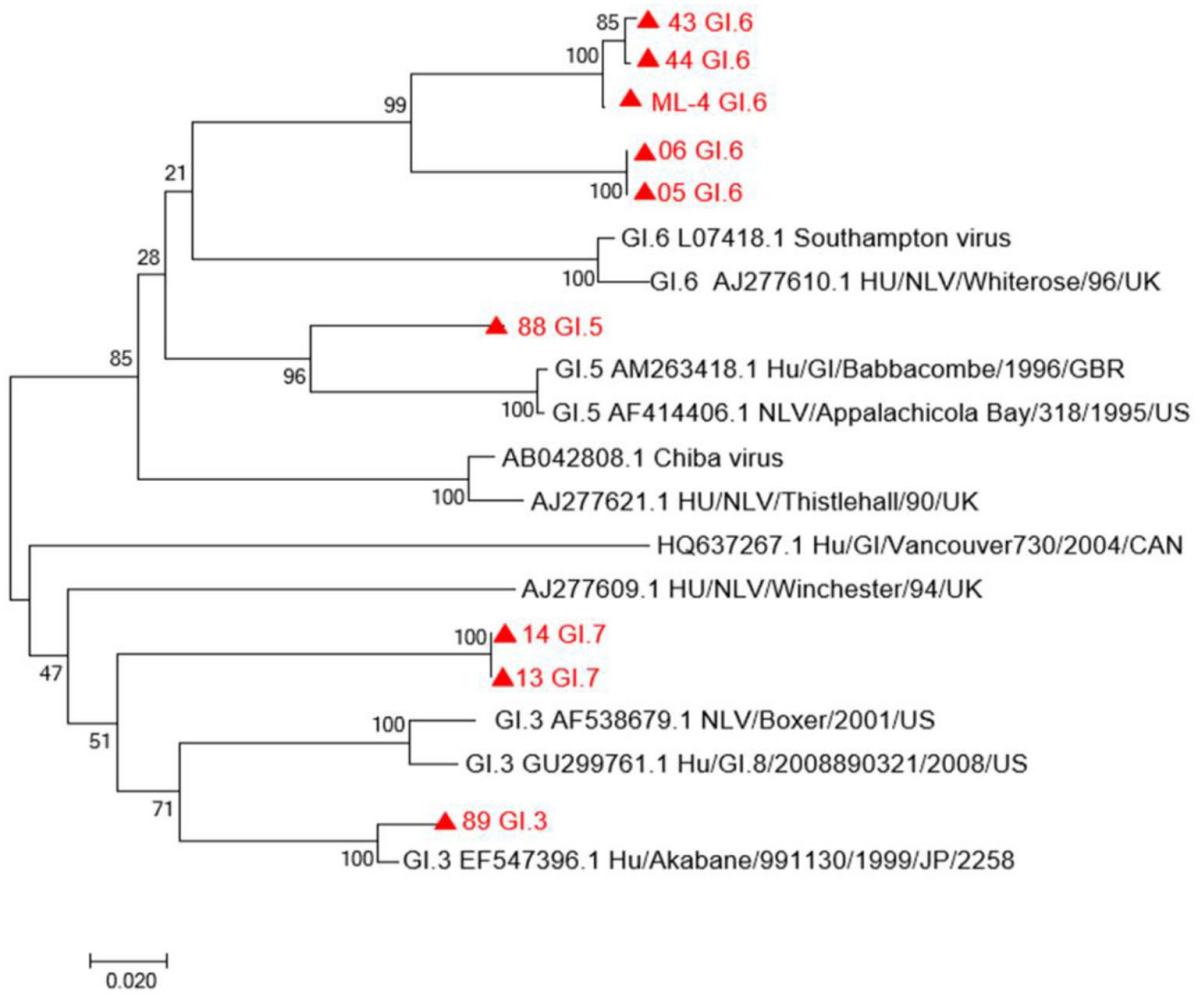
A phylogenetic tree of GI sequence in oysters and patients with diarrhea in Yantai, China. Virus strains indicated by 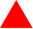 are from Yantai.

VP1 sequences of eight human NoV GI strains and the VP1 sequence of the oyster sample ML04 were used to construct a phylogenetic tree. As shown in Figure 2, from the analysis of the phylogenetic tree diagram, the ML04 sequence of one oyster belongs to the GI.6 type. Eight strains of human NoV contained GI.3, GI.5, GI.6, and GI.7. Among them, the samples No. 05, No. 06, No. 43, and No. 44 belong to GI. 6, and No. 43 and No. 44 belong to the same branch as oyster ML04. Ren88 belongs to GI.5, Ren13, and Ren14 belong to GI.7, and Ren89 belongs to GI.3.

### Homology Analysis of Oyster NoV GI Sequence and Human NoV GI Sequence in the Yantai Area

The nucleotide and amino acid sequences of eight human NoV GI strains in Yantai, Shandong province were analyzed, including GI.3, GI.5, GI.6, and GI.7. As shown in Table 4, the nucleotide and amino acid similarities of eight human NoV strains were 75.1–100% and 77.9–100%, respectively. Sequence numbers 05, 06, 43, and 44 belong to GI.6, and sample numbers 43 and 44 have the highest homology with ML04, with a nucleotide sequence similarity of 98.7% and an amino acid sequence similarity of 99.1%. The results indicated that the NoV sequences of human samples (Nos. 43 and 44) in Yantai, Shandong province were highly homologous to the ML04 sequences of oysters in Yantai.

**Table 4:** Nucleotide and amino acid homology analysis of the GI type in oysters and patients with diarrhea in Yantai, China.

**1**	**2**	**3**	**4**	**5**	**6**	**7**	**8**	**9**	
***	77.3	83.3	83.3	80.7	80.7	81.1	81.1	82	1.89 GI. 3
77.9	***	75.1	75.1	82.8	82.8	79.8	80.3	80.3	2.88 GI. 5
84.4	77.9	***	100	76	76	78.5	78.5	79	3.13 GI. 7
84.4	77.9	100	***	76	76	78.5	78.5	79	4.14 GI. 7
87	84.4	79.2	79.2	***	100	89.3	89.3	90.1	5.05 GI. 6
87	84.4	79.2	79.2	100	***	89.3	89.3	90.1	6.06 GI. 6
87	85.7	80.5	80.5	94.8	94.8	***	99.6	99.1	7.43 GI. 6
87	87	80.5	80.5	94.8	94.8	98.7	***	99.1	8.44 GI. 6
88.3	85.7	80.5	80.5	96.1	96.1	98.7	98.7	***	9. ML04 GI. 6

*Green is the nucleotide and Blue is the amino acid*.

## Discussion

In recent years, food safety accidents or public health emergencies caused by foodborne viruses have become increasingly prominent, which pose a serious threat to people's health and lives and become a major factor affecting social stability (12). NoV features low infection dose (10–100 virus particles), short incubation period (usually 12–48 h), long detoxification time (2–3 weeks), strong environmental resistance, and rapid transmission. Between 2000 and 2010, 36 outbreaks of acute viral gastroenteritis occurred worldwide, and 22 were related to eating shellfish contaminated by viruses, including NoV (13). In 2016, 75 cases of acute gastroenteritis caused by aquatic shellfish ingestion were found in Guangdong province, China. The detected NoV genotype was highly similar (95.8–99.7%) to GI.8 in the three kinds of samples (14). NoV GII17 was detected in oyster samples in Beijing between February 2014 and March 2015, which shared 99.2–100.0% similarity to the circulating human strain found in Japan between 2014 and 2015. GII.3 detected in the oyster samples in Beijing revealed 96.7% similarity with the circulating human strains in Beijing in 2015 while GII.4 detected in the oyster samples in Beijing exhibited 100% similarity with the prevalent human strains worldwide (16). Some researchers investigated the positive rate of asymptomatic infected persons and oysters with NoV in farms in southern China. The total infection rate of asymptomatic infected persons was 4.04%, and the incidence rate of NoV-infected cultured oyster population was 5.20%. The genotypes were diverse, GI.2, GI.3, GI.5, GI.6, GI.8, GII.3, GII.4, GII.5, GII.6, GII.8, GII.13, and GII.17 (17).

Studies have shown that oysters can serve as important carriers of NoV because their digestive gland cells have structures similar to NoV receptors, and NoV can be specifically enriched (18, 19). The results of this study showed that the positive rate of NoV in oysters in the Yantai area was much higher than that in other areas (20), indicating that oysters in this area were seriously polluted by NoV. From March to April 2019, the positive rate of NoV among patients with diarrhea in oyster gathering seafood markets in the same region was significantly higher than the national level during the same period (6). The high detection rate of NoV and the high incidence of associated diarrheal diseases may be related to the local habit of eating raw seafood.

Due to the low viral load in the majority of the collected oysters, only one GI-positive sequence was obtained in oysters, and 19 GII-positive sequences and eight GI-positive sequences were acquired in patients with diarrhea. To explore the possible correlation between oysters and patients with diarrhea, this study focused on the analysis of GI. Our phylogenetic analysis results showed that NoV sequences from oyster samples and some patients with diarrhea formed the same branch in the phylogenetic tree, belonging to GI.6, suggesting a close genetic relationship. As eight samples from GI-positive patients contained multiple types of GI.3, GI.5, GI.6, and GI.7, our results suggest that some patients with diarrhea in Yantai were caused by oyster consumption. In 2018, researchers detected NoV through the domestic sewage monitoring project in Jinan and Linyi cities, Shandong province, and found that GI.6 was the main epidemic type of urban domestic sewage and that the nucleotide sequences obtained from the sewage and patients were highly homologous at the same time in the same region, indicating that some transmission chains existed in these areas where NoV might circulate in humans, an aquatic environment, and shellfish (21). Several studies in Europe and Japan also mentioned the correlation between NoV pollution in the aquatic environment or shellfish and NoV infection in the human population (8–10). In two of the eight nororenia-related outbreaks reported by US researchers, the shellfish had 100% homology with the clinical strain (22). Our study provided additional evidence that NoV in the oyster was associated with human diarrhea in Yantai city, close to Jinan and Linyi cities. We speculated that the migration route is that the feces of NoV-infected people enter the water where the oysters continuously accumulate the virus. In turn, people may be infected with NoV through contact with NoV-contaminated water or eating NoV-contaminated oysters. Our study is the first report detecting NoV contamination in seafood sold in Yantai, Shandong province, which provides not only guidance to dealers and citizens consuming seafood, but also scientific data support for Yantai city to formulate food safety prevention strategies.

Our study has some limitations. First of all, the number of samples was not large enough to obtain the VP1 sequence from oysters, so the sample size should be increased in subsequent studies. Secondly, this study did not monitor and analyze NoV in Yantai seawater, epidemiological data of patients with diarrhea were not collected, and detailed information on the causes of the disease was not available. In the next step, we will address the detection and analysis of NoV in the offshore waters of Yantai and supplement epidemiological information related to patients with diarrhea for a better understanding of the circulating NoV infection path in Yantai, Shandong province.

## Conclusions

The results indicate that there may be a large number of NOVs circulating in the oyster population in Yantai city waters, which may cause seawater pollution and human infection with NoV, posing a health threat to the local population. We recommend that the local government should make more efforts to strictly monitor water quality in offshore aquatic markets and breeding areas, and enforce early preventive measures. Moreover, marketing staff should be educated to promote awareness about food safety; the management of a clean market environment should be strengthened to prevent cross-contamination of aquatic products; the circulation of aquatic products should be supervised to ensure clear traceability of goods sold on the market. Moreover, digestive tissues such as gills and intestines should be removed and thoroughly cooked when eating shellfish and seafood. At the same time, relevant departments should strengthen the supervision of markets for shellfish and seafood from different producing areas to assess the risks of consumption, which is critical to prevent NoV infection. Meanwhile, studies have shown that vaccines are the most cost-effective and effective means of preventing the disease. Due to the lack of *in vitro* cell culture systems and *in vivo* animal models for human NoV, no NoV vaccines have been used to prevent the occurrence of such diseases. However, there are ongoing efforts to develop a vaccine against NoV, and we hope that there will be a vaccine to prevent NoV in the near future.

## Data Availability Statement

The datasets presented in this study can be found in online repositories. The names of the repository/repositories and accession number(s) can be found below: GenBank, Accession Numbers ON042349 - ON042357.

## Ethics Statement

The study was conducted according to the guidelines of the Declaration of Helsinki, and approved by the Second Ethics Review Committee of the National Institute for Viral Disease Control and Prevention, Chinese Center for Disease Control and Prevention (Protocol Code IVDC2017NO.023 and DATE:4-21-2017). Written informed consent for participation was not required for this study in accordance with the national legislation and the institutional requirements.

## Informed Consent Statement

All patients read and signed informed consent.

## Author Contributions

JW and XM: data curation, project administration, and writing—review and editing. PN and MJ: formal analysis. ZS, PN, MJ, and QG: investigation. ZS, JW, and XM: methodology. MJ and QG: resources. ZS: writing—original draft. All authors contributed to the article and approved the submitted version.

## Funding

This work was funded by the Yantai Scientific Project, Grant No. 2017WS119.

## Conflict of Interest

The authors declare that the research was conducted in the absence of any commercial or financial relationships that could be construed as a potential conflict of interest.

## Publisher's Note

All claims expressed in this article are solely those of the authors and do not necessarily represent those of their affiliated organizations, or those of the publisher, the editors and the reviewers. Any product that may be evaluated in this article, or claim that may be made by its manufacturer, is not guaranteed or endorsed by the publisher.
